# Glucocorticoid‐Induced TNFR‐Related Ligand as a Biomarker for Diagnosis and Severity of Asthma in Children

**DOI:** 10.1002/pdi3.70020

**Published:** 2025-08-24

**Authors:** Yuehan Li, Yan Li, Jinying Xiang, Yinying Ren, Mi Zhou, Fang Deng, Zhou Fu, Fengxia Ding

**Affiliations:** ^1^ Department of Respiratory Medicine Ministry of Education Key Laboratory of Child Development and Disorders National Clinical Research Center for Child Health and Disorders China International Science and Technology Cooperation Base of Child Development and Critical Disorders Chongqing Engineering Research Center of Stem Cell Therapy Children's Hospital of Chongqing Medical University Chongqing China

**Keywords:** asthma, biomarker, children, GITRL

## Abstract

Currently, there are no reliable pediatric biomarkers for assessing the severity of asthma. Therefore, the study aimed to assess the potential of glucocorticoid‐induced TNFR‐related ligand (GITRL) in assessing asthma severity. Bone marrow‐derived dendritic cells (BMDCs) were isolated and treated with phosphate‐buffered saline (PBS) or house dust mites (HDM). Flow cytometry, immunofluorescence, and RT‐qPCR results revealed that GITRL expression was increased in HDM‐treated BMDCs (*p* < 0.05). An asthma mouse model was established using HDM. Asthmatic mice exhibited significantly increased lung inflammation scores, serum IgE, total cell counts, and eosinophil counts in bronchoalveolar lavage fluid (BALF) (*p* < 0.05). Tissue immunofluorescence and immunohistochemistry indicated elevated GITRL expression levels (*p* = 0.03). Flow cytometry analysis of peripheral blood mononuclear cells (PBMCs) isolated from children showed that the proportion of GITRL + dendritic cells (DCs) and the mean fluorescence intensity (MFI) of GITRL were significantly increased in asthmatic children compared with healthy individuals (*p* < 0.01). RT‐qPCR analysis showed that *GITRL* mRNA expression levels were significantly elevated in children with moderate‐to‐severe asthma compared to those with mild asthma (*p* = 0.006), and *GITRL* mRNA levels were higher in both groups of asthmatic children than in healthy controls (*p* < 0.05). Correlation analysis indicated that *GITRL* expression was negatively correlated with lung function and the Children's Asthma Control Test (C‐ACT) score (*p* < 0.001). ROC analysis assessed its diagnostic value, with AUC values of 0.89 (asthma vs. healthy diagnosis) and 0.70 (distinguishing moderate‐to‐severe from mild asthma). These findings suggest that GITRL is an important biomarker for assessing asthma severity.

## Graphical abstract

Our study demonstrates elevated GITRL expression in HDM‐exposed dendritic cells (in vitro), murine asthma models, and pediatric asthma patients, and reveals a direct association between GITRL levels and disease severity in children.

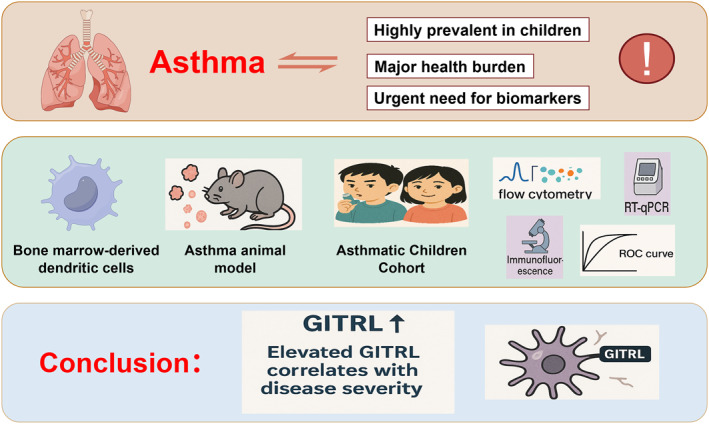

## 1 Introduction

Asthma is a widespread respiratory disease that currently affects an estimated 339 million individuals, with this number expected to rise to approximately 400 million by 2025 [1]. The Global Asthma Network reports a prevalence rate of 6.3% in children and 7.9% in adolescents, emphasizing its impact on pediatric health. These data underscore the urgency of identifying effective diagnostic and therapeutic strategies for childhood asthma [2].

Current guidelines consider lung function tests an important tool for diagnosing asthma. However, their applicability in pediatric populations, especially in preschool children, is significantly limited because of the difficulty in obtaining reliable data from repetitive exhalation tasks at this age [3, 4]. Therefore, in clinical practice, diagnosing asthma in children relies more heavily on symptom‐based criteria, including detailed medical history, recurrent wheezing episodes, and response to bronchodilators. Despite these diagnostic methods, the increasing incidence of severe pediatric asthma highlights the limitations of current strategies in early detection and personalized intervention [5]. In addition, first‐line treatments such as inhaled corticosteroids (ICSs) are effective in controlling inflammation, but they do not address the underlying causes of asthma and cannot cure the disease. Therefore, there is an urgent need to explore biomarkers related to the severity of airway inflammation and disease progression. Studying these biomarkers can not only improve diagnostic accuracy and disease monitoring but also provide new insights for the development of targeted treatments.

Asthma pathogenesis involves multiple cell types, including dendritic cells (DCs), T lymphocytes, B lymphocytes, eosinophils, mast cells, and neutrophils. They collectively contribute to the development of inflammation and airway hyperresponsiveness [6]. The interaction between DCs and T lymphocytes is central to this process because it could facilitate T cell activation [7]. This activation necessitates two signals: recognition of antigens via major histocompatibility complex class II (MHC II)‐T‐cell receptor (TCR) binding and co‐stimulatory signals provided by antigen‐presenting cells (APCs) [8]. Co‐stimulatory signaling molecules, as key mediators of DC maturation and immunoregulatory function, are an important component of DC‐dependent immunopathology in asthma. Among these, glucocorticoid‐induced TNFR‐related ligand (GITRL) is a key co‐stimulatory molecule because it can regulate immune responses by binding to the glucocorticoid‐induced TNF receptor (GITR) on T cells [9]. GITRL's expression is mainly limited to APCs, whereas GITR is widely expressed on T cells [10]. Evidence indicates that GITRL can enhance APC antigen presentation capabilities and influence T cell activation, proliferation, and differentiation [11, 12]. This signaling pathway can promote T cell differentiation into pro‐inflammatory phenotypes such as T helper type 2 (Th2) cells and T helper 17 (Th17) cells, whereas decreased GITRL expression may promote T helper type 1 (Th1) cells polarization [13, 14]. Such immune imbalances are closely linked to asthma pathophysiology. Despite its importance, its application as a diagnostic–prognostic biomarker in asthma remains underexplored, especially in pediatric populations.

Based on the above background, we hypothesized that GITRL levels were increased in asthmatic children and were positively associated with disease severity. This investigation aims to explore the function of GITRL in pediatric asthma and to evaluate its practicality as a biomarker for disease diagnosis and severity assessment in clinical practice.

## 2 Methods

### 2.1 Approval From Ethics Committees

The study plan was approved by the local ethics committee (Animal Approval No. CHCMU‐IACUC20241018007, Human Approval No. 2022.373). All procedures adhered to the IACUC guidelines for animal experiments and complied with the Helsinki Declaration for human research. This study was registered on ClinicalTrials.gov (NCT05768399), with documented informed consent secured from the guardians of participating children.

### 2.2 Cell Culture

BMDCs were generated from the femurs and tibiae of C57BL/6 mice. The cells were grown under granulocyte‐macrophage colony‐stimulating factor (GM‐CSF, 20 ng/mL) and interleukin‐4 (IL‐4, 10 ng/mL) in RPMI‐1640/10% fetal bovine serum (FBS). After 6 days, cells were harvested for subsequent experimental use.

### 2.3 Cell Immunofluorescence

BMDCs were cultured in confocal petri dishes and exposed to house dust mites (HDM) or phosphate‐buffered saline (PBS). After treatment, the cells underwent fixation, permeabilization, and blocking. Cells were treated with primary antibodies and incubated overnight: anti‐CD11c‐FITC (green, 1:50, Becton Dickinson & Company, Mountain View, California, USA) for DCs, anti‐GITRL (red, 1:100, GeneTex, Irvine, California, USA) for GITRL expression, and DAPI (blue, 1:100, Beyotime, Shanghai, China) for nuclei. After washing, Alexa Fluor 594‐conjugated secondary antibody was applied to cells for 1 h followed by observation with a confocal fluorescence microscope (Zeiss, Oberkochen, Germany).

### 2.4 Asthma Mouse Model

Specific pathogen‐free (SPF)‐grade female C57BL/6 mice were obtained from Experimental Animal Facility of Chongqing Medical University. After a 7‐day acclimation, 10 mice were split into control (*n* = 5) and asthma (*n* = 5) groups. Following established protocols, mice were sensitized/challenged with HDM (Greer Laboratories, Lenoir, North Carolina, USA) extract via intranasal instillation [15], whereas the control group received saline alone. After that, euthanasia was performed under isoflurane anesthesia for specimen collection.

### 2.5 Bronchoalveolar Lavage and Cell Enumeration

Lungs from each mouse were lavaged with cold PBS to obtain bronchoalveolar lavage fluid (BALF). Total cell numbers were obtained by counting with a hemocytometer. Subsequently, smears were processed using a cytospin and then stained with Wright–Giemsa (Solarbio, Beijing, China). Following this, eosinophils were quantified under a light microscope.

### 2.6 Lung Histopathology

Lung samples from mice were preserved in 4% paraformaldehyde, paraffin‐embedded, sliced, and subsequently stained with hematoxylin and eosin (H&E). Then we employed the Peebles RS method, which evaluates airway inflammation in the trachea, bronchioles, perivascular regions, and pulmonary interstitium [15]. Inflammation in the peribronchial and peribronchiolar regions, as well as in perivascular areas and the pulmonary interstitium, was scored based on the thickness of inflammatory cell infiltrates. Peribronchial and perivascular inflammation was assessed as follows: 0 (no infiltration), 1 (≤ 2 cell layers for bronchioles, ≤ 4 cell layers for vessels), 2 (3–5 layers for bronchioles, 5–7 layers for vessels), and 3 (> 5 layers for bronchioles, > 7 layers for vessels). Pulmonary interstitial inflammation was classified as 0 (absence of infiltration), 1 (infiltration without septal thickening), 2 (infiltration with mild thickening), and 3 (severe infiltration with severe thickening).

### 2.7 Immunohistochemistry

Lung tissue sections embedded in paraffin were subjected to deparaffinization and heat‐assisted antigen retrieval was performed, and endogenous peroxidase activity was blocked using 3% hydrogen peroxide (H₂O₂). Blocking was performed using 5% bovine serum albumin (BSA). Sections were incubated with a primary GITRL antibody diluted 1:200 (GeneTex, Irvine, California, USA). Then the sections were treated with an horseradish peroxidase (HRP)‐labeled secondary antibody. Visualization was achieved using diaminobenzidine (DAB) as the chromogen, then the slides underwent hematoxylin counterstaining before mounting.

### 2.8 Participants

A total of 60 pediatric patients with clinically diagnosed asthma and 49 healthy controls were recruited. The study initially enrolled 17 asthmatic children and 13 healthy controls (Group 1) to explore differences in GITRL expression between asthmatic and non‐asthmatic populations. To further investigate the association between GITRL and asthma severity, we expanded the sample size by including children with both mild and moderate‐to‐severe asthma. This led to the recruitment of the second independent cohort (Group 2), consisting of 43 asthmatic children (23 mild, 20 moderate‐to‐severe) and 36 additional healthy controls.

**FIGURE 1:**
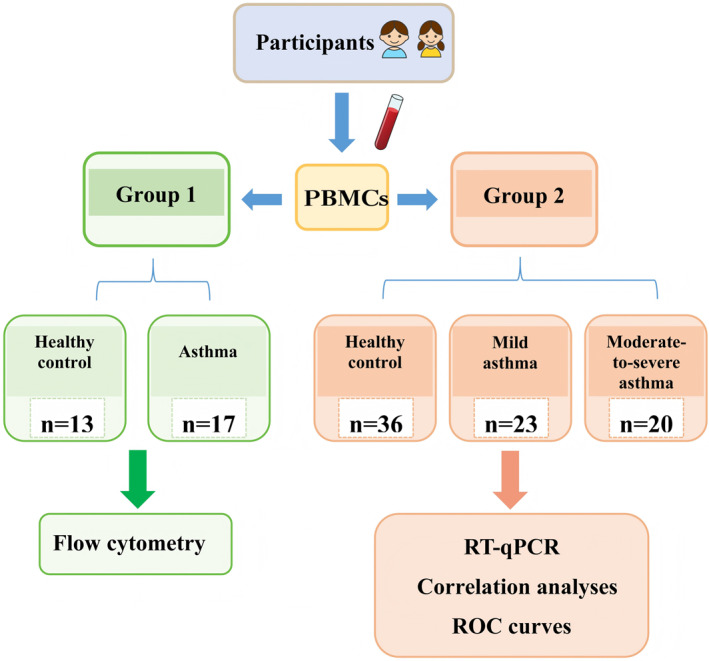
The flow diagram of the study. PBMCs, peripheral blood mononuclear cells.

Participant selection adhered to inclusion and exclusion criteria specified in Table S1. Asthma diagnoses were confirmed by board‐certified pediatric pulmonologists, and severity was graded according to the Global Initiative for Asthma (GINA) guidelines [16]. Peripheral blood samples were obtained from each participant, and demographic data, clinical features, and childhood asthma control test (C‐ACT) questionnaire responses were simultaneously recorded. A detailed recruitment and study workflow is illustrated in Figure 1.

### 2.9 Peripheral Blood Mononuclear Cells (PBMCs) Isolation

Separate peripheral blood mononuclear cells using the Ficoll–Paque density gradient method (Hao Yang Bio, Tianjin, China). Peripheral blood samples (2 mL) were collected aseptically using EDTA‐coated tubes. After diluting 1:1 with PBS, the whole blood was carefully layered over the gradient medium. Following centrifugation at 500 × g for 30 min, the PBMC layer (second interface) was obtained, followed by PBS washing, and then resuspended for subsequent analyses.

### 2.10 Flow Cytometry

PBMCs were pre‐blocked with rat serum and then stained with fluorescently labeled antibodies, including anti‐CD11c‐PerCP‐Cy5.5 (Biolegend, San Diego, California, USA), anti‐MHC II‐APC/Cyanine7 (Biolegend, San Diego, California, USA), and anti‐GITRL‐Alexa Fluor 647 (Abcam, Cambridge, UK). Data collection was conducted using a BD FACSCanto II instrument and analyzed via FlowJo software. Compensation and gating strategies were standardized across all samples.

### 2.11 RT‐qPCR

Total RNA was isolated and quantified, followed by cDNA synthesis and RT‐qPCR, and relative expression was determined via the 2^−ΔΔCt^ method. Primer sequences included the following Table 1.

**TABLE 1:** Primer sequences used for RT‐qPCR analysis.

Gene(species)	Forward(5′–3′)	Reverse(5′–3′)
*GITRL* (Mouse)	TCGAGTCCTGCATGGTTAAGT	ACTACGAAGGGGGCATTGTC
*β‐actin* (Mouse)	CATCCGTAAAGACCTCTATGCCAAC	ATGGAGCCACCGATCCACA
*GITRL* (Human)	GCTCAGAGATCATCCTGGAAGC	TAGCCATACAGGGCTCCTTAGC
*GAPDH* (Human)	AATGGGCAGCCGTTAGGAAA	GCGCCCAATACGACCAAATC

*Note:* Primer orientation is indicated as forward and reverse (5′–3′). Mouse *β‐actin* and human *GAPDH* were used as housekeeping genes for normalization.

### 2.12 Statistical Analyses

Data analysis was performed using SPSS Statistics 27.0 (IBM) and GraphPad Prism 8.0. For comparisons between two independent continuous variables, independent‐sample t‐tests were employed, whereas one‐way ANOVA was used for multiple group comparisons. Categorical variables were analyzed using the Chi‐squared test or Fisher's exact test. Spearman's correlation and receiver operating characteristic (ROC) curve analyses were performed for exploratory purposes. *p* < 0.05 was considered significant.

## 3 Results

### 3.1 Elevated GITRL Levels in BMDCs Induced by HDM Exposure

**FIGURE 2:**
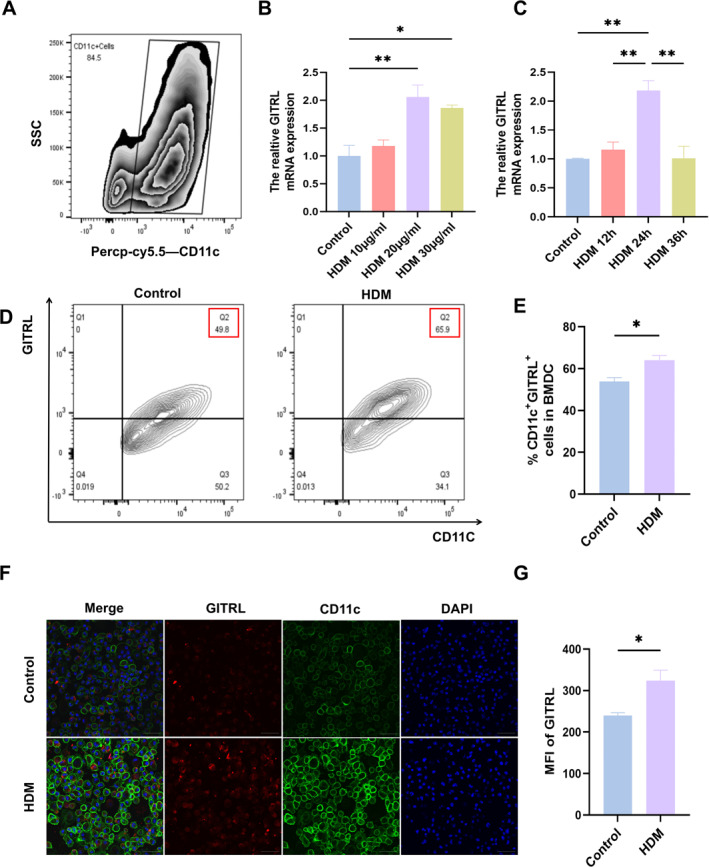
GITRL levels in BMDCs treated with HDM/PBS. (A) Flow cytometry analysis confirming the purity of BMDCs. (B) *GITRL* expression levels in BMDCs treated with various concentrations of HDM for 24 h, assessed by RT‐qPCR. (C) *GITRL* expression levels in BMDCs exposed to HDM (20 μg/mL) for different durations, measured by RT‐qPCR. (D) GITRL expression levels in BMDCs as determined by flow cytometry. (E) Comparison of CD11c + GITRL + cells in BMDCs treated with PBS or HDM. (F) GITRL expression levels in BMDCs detected by immunofluorescence. (G) Comparison of the mean fluorescence intensity (MFI) of GITRL in BMDCs treated with PBS or HDM. (**p < 0.01; *p < 0.05).

BMDCs, purified to over 80% from C57BL/6 mice (Figure 2A), were exposed to various concentrations of HDM for defined time intervals. Analysis by RT‐qPCR demonstrated a significant elevation in *GITRL* mRNA expression, peaking at 20 μg/mL HDM after 24 h (Figure 2B, *p* = 0.006, Figure 2C, *p* = 0.002). Flow cytometry analysis identified a marked increase in CD11c + GITRL + cell populations in HDM‐treated BMDCs compared to PBS controls (Figure 2D,E, *p* = 0.01). Immunofluorescence staining confirmed these findings, highlighting the upregulation of GITRL upon HDM stimulation (Figure 2F,G, *p* = 0.03). Collectively, these results suggest that HDM exposure triggers a significant increase in GITRL expression in BMDCs.

### 3.2 Upregulated GITRL Levels in an HDM‐Driven Asthma Model

**FIGURE 3:**
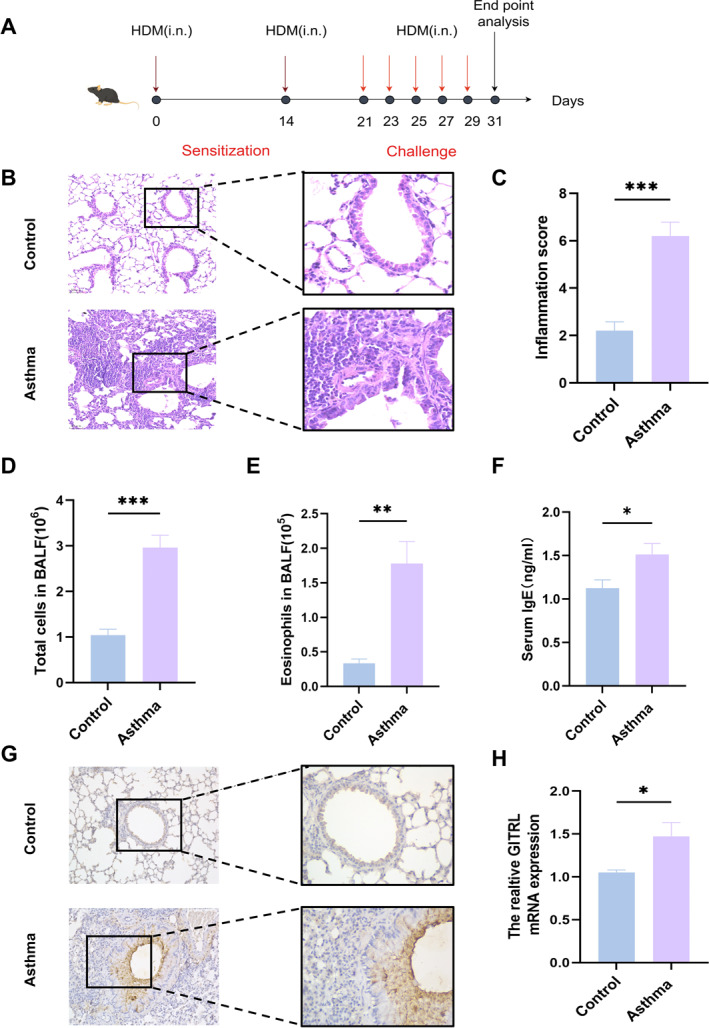
Expression of GITRL in the lungs of control and asthmatic mice. (A) Schematic representation of the protocol used for the murine asthma model. (B) H&E staining of lung sections. (C) Comparison of inflammation scores between control and asthmatic mice. (D) Comparison of total cell counts in the BALF between control and asthmatic mice. (E) Comparison of eosinophil counts in the BALF between control and asthmatic mice. (F) Serum levels of total IgE in control and asthmatic mice, measured by enzyme‐linked immunosorbent assay (ELISA). (G) Levels of GITRL in the lungs of control and asthmatic mice, assessed by immunohistochemistry. (H) *GITRL* expression levels in the lungs of control and asthmatic mice, as determined by RT‐qPCR. (***p < 0.001; **p < 0.01; *p < 0.05).

An asthma model was established via repeated HDM exposure (Figure 3A). Histological analysis of lung tissues from asthmatic mice revealed extensive inflammatory cell infiltration, peribronchial and perivascular thickening, and increased interstitial inflammation compared to PBS controls (Figure 3B,C, *p* < 0.001). BALF analysis indicated elevated total cell counts (Figure 3D, *p* < 0.001), eosinophil proportions (Figure 3E, *p* = 0.002), and serum IgE levels (Figure 3F, *p* = 0.04) in asthmatic mice. Immunohistochemistry and RT‐qPCR confirmed an upregulation of GITRL levels in the lungs of HDM‐exposed mice compared to controls (Figure 3G,H, *p* = 0.03).

### 3.3 Elevated GITRL Levels in Pediatric Asthma Compared to Healthy Controls

**FIGURE 4:**
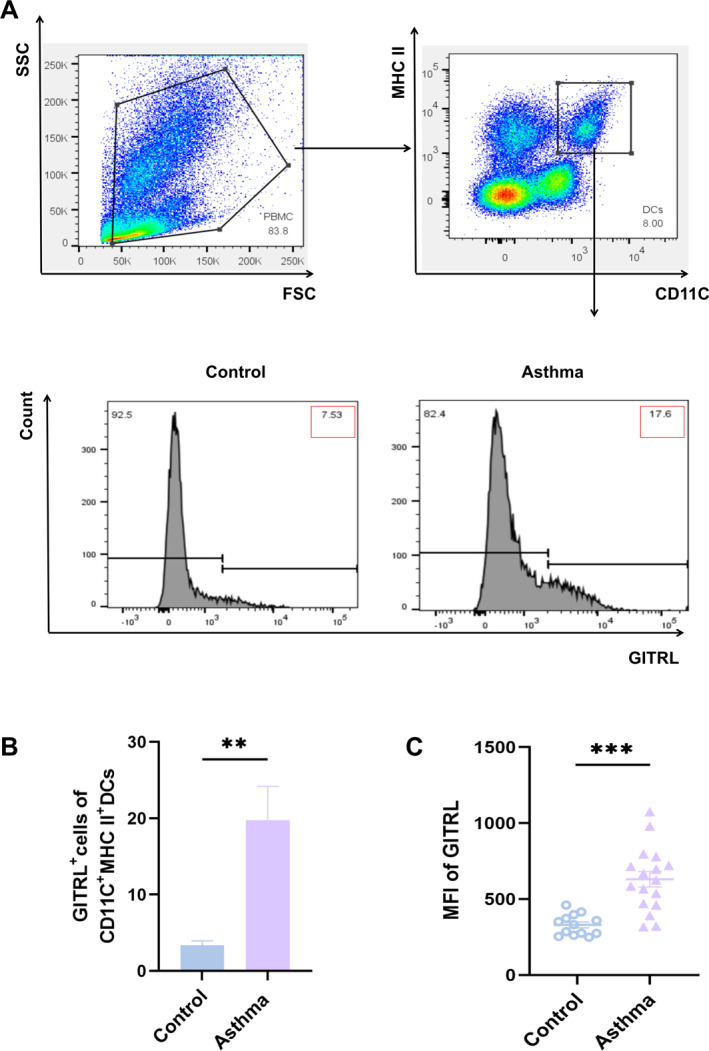
GITRL expression levels in dendritic cells of children with asthma. (A) Detection of GITRL expression in dendritic cells using flow cytometry. (B) Comparison of the proportion of GITRL + CD11c + MHC II + dendritic cells in healthy controls versus children with asthma. (C) Comparison of the mean fluorescence intensity of GITRL in healthy controls and children with asthma. (***p < 0.001; **p < 0.01).

To investigate the differences in GITRL expression in DCs derived from PBMCs between asthmatic children and healthy controls, we included 17 children with clinically diagnosed asthma and 13 healthy counterparts (Group 1). Table 2 presents comprehensive demographic and clinical information. Asthmatic children exhibited significantly lower lung function parameters, such as forced expiratory volume in 1 s (FEV1, *p* < 0.001), forced vital capacity (FVC, *p* < 0.001), FEV1/FVC% (*p* = 0.003), and peak expiratory flow (PEF, *p* < 0.001), compared to healthy subjects. Flow cytometry analysis was performed to quantify the level of GITRL in CD11c + MHC II + dendritic cells (Figure 4A; gating strategy and isotype controls detailed in Figure S1). The results demonstrated a notable rise in both the percentage of GITRL‐expressing DCs (Figure 4B, *p* = 0.004) and the mean fluorescence intensity (MFI) of GITRL (Figure 4C, *p* < 0.001) in asthmatic children compared to healthy controls. These findings underscore an upregulation of GITRL in asthma, indicating its possible role in the underlying immune dysregulation.

**TABLE 2:** Clinical characteristics of Group 1.

	Healthy control (*n* = 13)	Asthma (*n* = 17)	*p* value
Sex (male/female), no. (%)	5 (38)/8 (62)	8 (47)/9 (53)	0.64
Age (years)	7.5 ± 0.7	7.1 ± 0.6	0.65
BMI (kg/m^2^)	15.5 ± 0.4	15.4 ± 0.4	0.88
Pulmonary function test			
FEV1 (% predicted)	113.1 ± 3.6	85.4 ± 1.8	< 0.001
FVC (% predicted)	104.8 ± 3.1	88.1 ± 2.4	< 0.001
FEV1/FVC (%)	92.6 ± 1.1	83.7 ± 2.2	0.003
PEF (% predicted)	102.2 ± 3.2	78.1 ± 2.1	< 0.001

Abbreviations: BMI: Body mass index; FEV1: Forced expiratory volume in 1 s; FVC: Forced vital capacity; PEF: Peak expiratory flow.

### 3.4 Higher GITRL Expression Corresponds to Increased Asthma Severity

**FIGURE 5:**
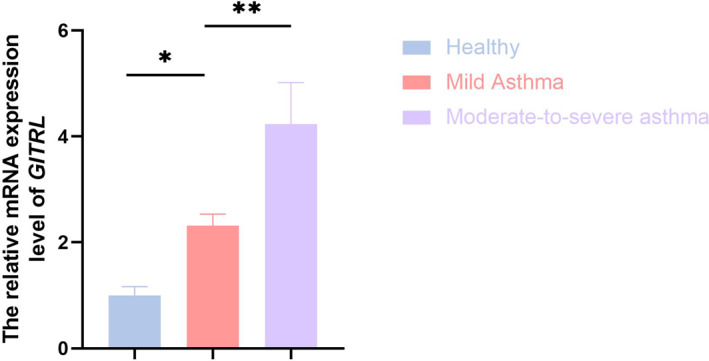
Relative mRNA expression levels of *GITRL* in PBMCs across three groups: healthy controls, children with mild asthma, and children with moderate‐to‐severe asthma. Inter‐group comparisons revealed significant differences in GITRL expression levels, with statistical significance indicated by *p < 0.05 and **p < 0.01.

We expanded the scope of our study to include 43 children with asthma, 23 of whom had mild asthma, 20 had moderate‐to‐severe asthma, and 36 were healthy controls (Group 2). The characteristics of all participants are listed in Table 3. Children with asthma—especially those with moderate‐to‐severe disease—exhibited poorer lung function (*p* < 0.001) and higher rates of allergic comorbidities (*p* < 0.001) and family history of asthma (*p* = 0.04). Using RT‐qPCR, we measured *GITRL* mRNA levels in PBMCs. Data showed that the level of GITRL was significantly higher in asthmatic children than healthy controls (*p* = 0.04), with even greater elevations observed in those with moderate‐to‐severe asthma (*p* = 0.006) (Figure 5). This trend indicates a positive correlation between GITRL expression and asthma severity, potentially highlighting its role in disease progression.

**TABLE 3:** Clinical characteristics of Group 2.

	Healthy control (*n* = 36)	Mild asthma (*n* = 23)	Moderate‐to‐severe asthma (*n* = 20)	*p* value
Sex (male/female), no. (%)	17 (47)/19 (53)	13 (57)/10 (43)	12 (60)/8 (40)	0.61
Age (years)	7.4 ± 0.3	6.3 ± 0.4	6.4 ± 0.5	0.05
BMI (kg/m^2^)	15.9 ± 0.4	16.1 ± 0.7	15.7 ± 0.6	0.89
Smoke exposure, no. (%)	15 (41.7)	13 (57)	13 (65)	0.21
Pets at home, no. (%)	8 (22.2)	4 (17.4)	2 (8.7)	0.52
Breast‐feeding, no. (%)	26 (72.2)	18 (78.3)	11 (55)	0.25
Atopy, no. (%)	6 (16.7)	18 (78.3)^a^	18 (90.0)^a^	< 0.001
Food allergy sensitizations, no. (%)	5 (13.9)	5 (21.7)	6 (30.0)	0.69
Aeroallergen sensitizations, no. (%)	3 (8.3)	16 (70)^a^	17 (85)^a^	< 0.001
Family history of asthma, no. (%)	2 (5.6)	5 (21.7)	6 (30)^a^	0.04
Pulmonary function test				
FEV1 (% predicted)	111.5 ± 2.3	88.5 ± 1.3^a^	65.5 ± 2.6^a^ ^,^ ^b^	< 0.001
FVC (% predicted)	103.7 ± 2.1	91.3 ± 1.9^a^	74.5 ± 3.0^a^ ^,^ ^b^	< 0.001
FEV1/FVC (%)	92.1 ± 0.6	83.6 ± 1.8^a^	75.3 ± 1.6^a^ ^,^ ^b^	< 0.001
PEF (% predicted)	105.6 ± 3.1	83.4 ± 1.6^a^	63.0 ± 3.4^a^ ^,^ ^b^	< 0.001
C‐ACT	/	24.5 ± 0.4	18.5 ± 0.8	< 0.001

Abbreviations: BMI: Body mass index; C‐ACT: Childhood asthma control test; FEV1: Forced expiratory volume in 1 s; FVC: Forced vital capacity; PEF: Peak expiratory flow.

^a^ *P* < 0.05 in mild or moderate‐to‐severe asthma versus healthy control group.

^b^ *P* < 0.05 in moderate‐to‐severe asthma versus mild asthma group.

### 3.5 Correlation Between GITRL Expression, Lung Function, and C‐ACT Scores

**FIGURE 6:**
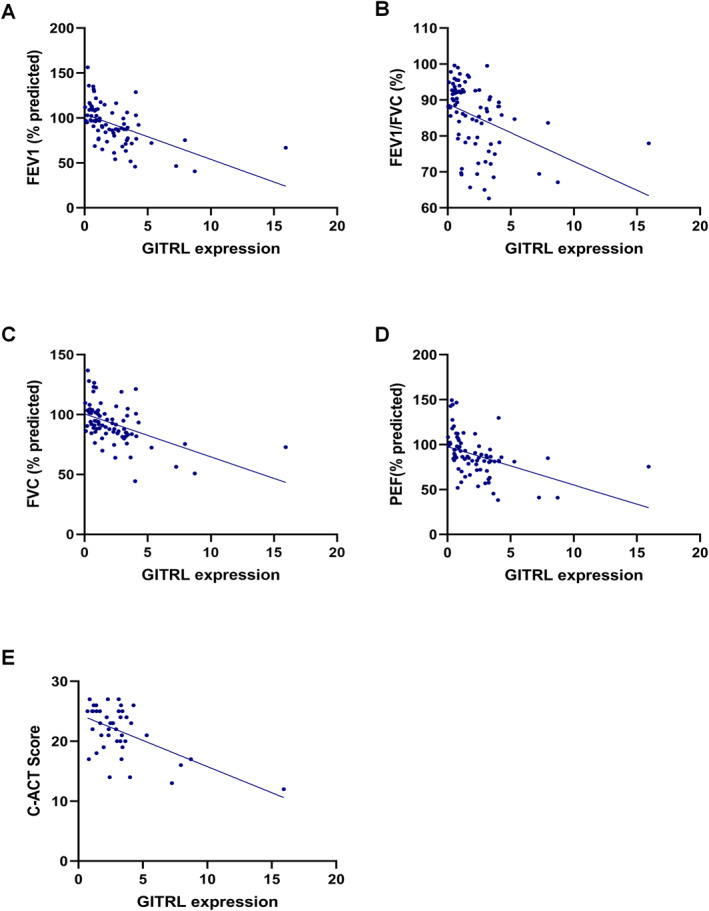
Spearman correlation analysis of GITRL expression levels with lung function parameters and C‐ACT scores. (A) Correlation between GITRL expression levels and predicted FEV1% (*p* < 0.001). (B) Correlation between GITRL expression levels and the FEV1/FVC ratio (*p* < 0.001). (C) Correlation between GITRL expression levels and predicted FVC% (*p* < 0.001). (D) Correlation between GITRL expression levels and predicted PEF% (*p* < 0.001). (E) Correlation between GITRL expression levels and C‐ACT scores (*p* < 0.001).

Next, we conducted correlation analyses between GITRL expression and clinical parameters of asthma severity. We found that GITRL levels were inversely correlated with FEV1% (*r* = −0.549, *p* < 0.001), FEV1/FVC ratio (*r* = −0.519, *p* < 0.001), FVC% (*r* = −0.629, *p* < 0.001), and PEF% (*r* = −0.611, *p* < 0.001) (Figure 6A–D). Similarly, a negative association was observed between GITRL expression and C‐ACT scores (*r* = −0.408, *p* < 0.001, Figure 6E). These results demonstrate that higher GITRL expression corresponds to greater asthma severity, further confirming its potential as a marker for assessing disease control and lung function impairment.

### 3.6 GITRL Expression as a Diagnostic and Prognostic Biomarker for Asthma

**FIGURE 7:**
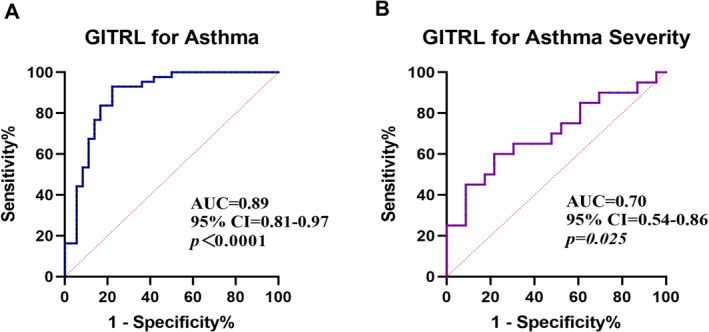
GITRL expression as a biomarker for asthma and its severity. (A) ROC curve analysis for predicting asthma based on GITRL expression (*p* < 0.001). (B) ROC curve analysis for distinguishing moderate‐to‐severe asthma from mild asthma using GITRL expression (*p* = 0.025). AUC, area under the curve; CI, confidence interval.

To assess the diagnostic and prognostic value of GITRL expression in asthma, we performed ROC curve analysis. The diagnostic capability of GITRL for distinguishing asthmatic children from healthy controls yielded an AUC of 0.89 (*p* < 0.001) (Figure 7). When evaluating its ability to differentiate mild asthma from moderate‐to‐severe asthma, the AUC was 0.70 (*p* = 0.025). Our findings indicate that GITRL expression potentially acts as a promising biomarker not only for identifying asthma but also for assessing its severity, which may aid clinical decision‐making and the development of personalized treatment strategies.

## 4 Discussion

Our research shows that GITRL is highly expressed in mouse asthma models and pediatric asthma patients, supporting its potential role in asthma immunopathogenesis. It is worth noting that GITRL levels in children with moderate to severe asthma were significantly higher than those in children with mild disease, indicating a correlation between GITRL expression and asthma severity. Furthermore, the negative correlation between GITRL levels and pulmonary function parameters or C‐ACT scores further confirms its practicality as a biomarker for assessing severity. GITRL has high diagnostic accuracy (AUC 0.89) and moderate severity discrimination ability (AUC 0.70), making it a promising translational medicine tool, especially in pediatric cases where traditional pulmonary function testing is challenging.

Consistent with our findings implicating GITRL in promoting asthmatic inflammation, its pro‐inflammatory properties have been confirmed in a variety of immune‐related diseases. For example, in atherosclerosis, GITRL exacerbates vascular inflammation by upregulating tumor necrosis factor‐α (TNF‐α) and matrix metalloproteinase‐9 (MMP‐9) expression, thereby accelerating plaque progression [17]. Similarly, elevated GITRL levels in rheumatoid arthritis patients are positively correlated with systemic inflammatory burden [18]. In acute respiratory distress syndrome (ARDS), GITRL upregulation under inflammatory stimuli can be suppressed by dexamethasone through regulation of the GITR/GITRL axis [19]. It is worth noting that although these studies collectively emphasize GITRL's role in enhancing immune activation, its involvement in asthma—especially in clinical settings—remains to be fully explored. Our study fills this gap, demonstrating for the first time a direct correlation between GITRL expression and asthma severity.

Unlike previous studies, this study focuses specifically on the expression of GITRL in DCs in the context of asthma. As a tumor necrosis factor receptor family member, GITRL may participate in the development of T2‐type inflammation by regulating antigen presentation mediated by DCs. Although the mechanism of asthma requires further study, previous studies have shown that GITRL can affect T cell differentiation. It has been observed that it alters the balance between Th17 cells and regulatory T cells (Tregs) in autoimmune conditions (such as autoimmune thyroiditis [20]and Sjogren's syndrome [21]). Similarly, in the field of oncology, GITRL is shown to be linked to enhanced antitumor immunity via activating effector T cells and suppressing Treg function [22, 23]. Given the key role of CD4+ T cell subsets in the asthma pathophysiology, GITRL may be involved in regulating immune responses, thereby potentially influencing the severity of asthma.

The causes of asthma are not yet fully understood [24]. Over recent decades, worldwide asthma rates, especially among children, have been on the rise, leading to increased medical costs and a heavier social burden [25, 26]. Severe asthma cases constitute more than half of all asthma‐related healthcare expenses. At the same time, their persistence and significant risk of complications are particularly worrying [27]. Uncontrolled asthma can lead to adverse clinical outcomes, such as airway remodeling, respiratory failure, and accompanying neuropsychiatric disorders, including mood disorders and specific cognitive impairments [28, 29]. These complexities highlight the necessity of early identification and the development of personalized intervention strategies.

Although our understanding of asthma has improved, its pathogenesis remains unclear. Immune dysregulation is increasingly recognized as center to asthma development, with APCs, especially DCs, playing a crucial role [30]. DCs are potent APCs that connect innate and adaptive immune responses [31]. Upon maturation, DCs promote T cell differentiation toward Th2 and Th17 dominance, which is pivotal in the inflammatory cascade of asthma [32]. The maturation process of DCs is characterized by the upregulation of MHC ELISA II and co‐stimulatory molecules [33]. These co‐stimulatory molecules not only serve as markers of DC maturation but also function as key mediators of immune responses. Therefore, co‐stimulatory molecules represent important potential therapeutic targets in asthma management [34, 35].

Primarily, GITR/GITRL signaling regulates T cell responses through NF‐κB and MAPK pathways. GITR recruits the TRAF family, among which TRAF2, TRAF5, and TRAF6 play key roles in T cell function [36]. The co‐stimulatory signal of GITR activates the NF‐κB signaling pathway through TRAF6, influencing CD4+ T cell differentiation and enhancing effector T cell function [37]. The GITR–GITRL signaling pathway also regulates immune responses through its impact on the activity and migratory function of DCs, as well as the regulation of cytokine secretion [13].

Compared with traditional biomarkers (such as FeNO and blood eosinophils), GITRL is more directly associated with T‐cell immune responses. It can also more directly reflect the potential immune inflammatory state in asthma patients. Furthermore, whereas FeNO and eosinophil counts are mainly used to identify and evaluate type 2 (T2) inflammation, GITRL expression analysis is applicable to both T2 and non‐T2 asthma phenotypes, demonstrating broader applicability. In the field of treatment, anti‐GITRL monoclonal antibodies have entered phase I clinical trials in oncology [38]. In animal models, the GITR‐Fc fusion protein has been shown to have clear efficacy by competitively binding to GITRL [39]. These precedents highlight the translational medical potential of targeting GITRL for asthma treatment, further confirming its enormous potential for clinical translation. Based on our research findings, future studies could explore the clinical translation of GITRL through the following approaches: Validate its correlation with asthma severity and prognosis through a multicenter clinical cohort study, subsequently assess the therapeutic potential of targeted treatment strategies in non‐human primate animal models, and finally explore its potential application in the clinical management of asthma patients.

## 5 Conclusion

In this study, we reveal that GITRL, a member of the co‐stimulatory molecule family, shows significant elevation in pediatric asthma and correlates with disease severity. We have demonstrated that GITRL is an effective diagnostic biomarker for asthma. However, further investigation is required to elucidate the mechanisms by which GITRL influences asthma progression, thereby providing new strategies for disease management.

## Author Contributions

**Yuehan Li:** conceptualization, data curation, formal analysis, investigation, methodology, writing – original draft, writing – review and editing. **Yan Li:** data curation, formal analysis, methodology, writing – original draft. **Jinying Xiang:** conceptualization, investigation, methodology, validation, writing – review and editing. **Yinying Ren:** data curation, formal analysis and writing – original draft. **Mi Zhou:** conceptualization, investigation, methodology, validation, writing – review and editing. **Fang Deng:** data curation, formal analysis and writing – original draft. **Zhou Fu:** project administration, resources and supervision. **Fengxia Ding:** conceptualization, funding acquisition, methodology, project administration, resources, supervision, validation, writing – review and editing.

## Ethics Statement

The animal study protocol was approved by the Ethics Committee of the Children's Hospital of Chongqing Medical University (Approval No. CHCMU‐IACUC20241018007). The human study was conducted in accordance with the Declaration of Helsinki and approved by the same committee (Approval No. 2022.373).

## Conflicts of Interest

The authors declare no conflicts of interest.

## Supporting information


Supporting Information S1

## Data Availability

The data that support the findings of this study are available from the corresponding author upon reasonable request.
