# Supplementary material for: Glucocorticoid‐Induced TNFR‐Related Ligand as a Biomarker for Diagnosis and Severity of Asthma in Children
Source: Pediatr Discov. 2025 Aug 24;4(1):e70020. doi: 10.1002/pdi3.70020 (PMC13098139; doi:10.1002/pdi3.70020)
Supplement: Supplementary file 1 — Supporting Information S1 [file PDI3-4-e70020-s001.docx]

**Supplementary Figure 1.** Gating strategy and isotype controls for CD11c+MHCII+ DCs.


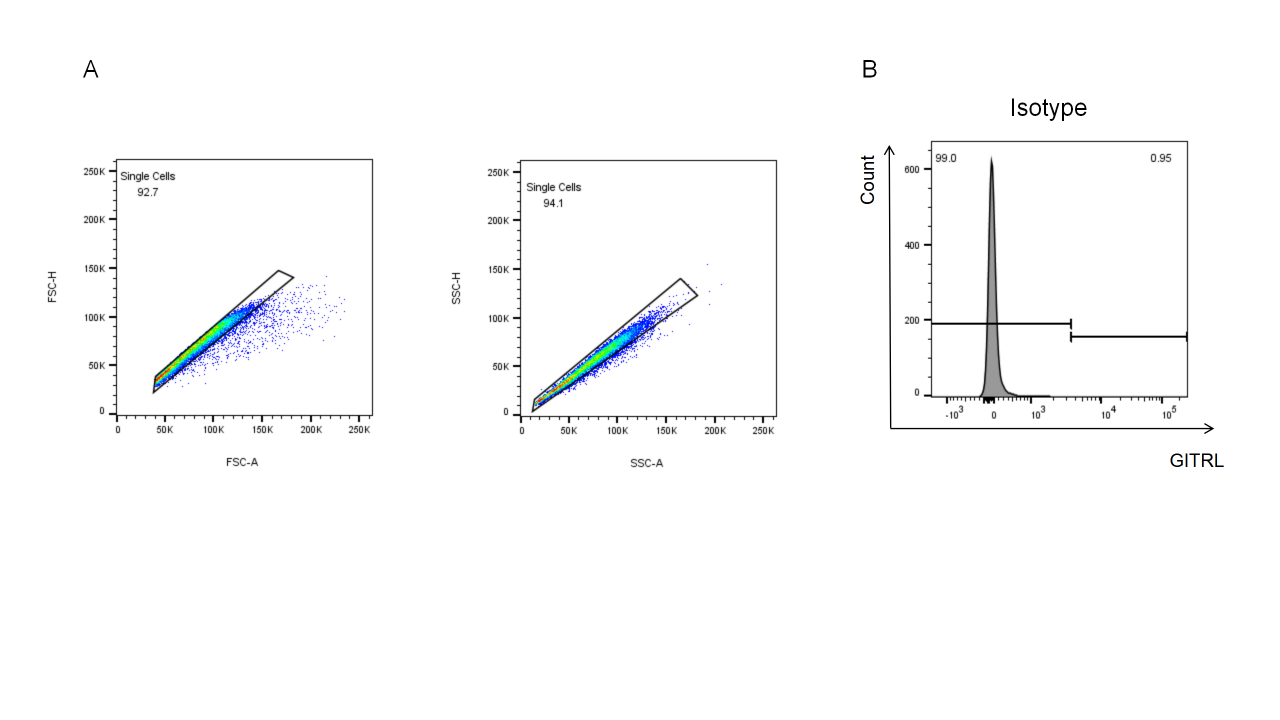


Gating strategy(A) and isotype controls(B) for CD11c+MHCII+ DCs.

**Supplementary Table 1.** Participant selection adhered to inclusion and exclusion criteria.

| Inclusion Criteria | a) Diagnosis of asthma in accordance with the latest guidelines from the Global Initiative for Asthma (GINA).  b) Age: 4-12 years.  c) Typical clinical manifestations and comprehensive clinical data available.  d) Informed consent obtained and signed by the legal guardians. |
| --- | --- |
| Exclusion Criteria | a) Presence of other respiratory diseases (such as bronchiectasis, respiratory failure, pulmonary tuberculosis, or airway obstructive diseases) or inflammatory conditions in other body parts.  b) Congenital anomalies of the airways or lung tissue.  c) Administration of immunosuppressants or corticosteroids within 4 weeks prior to enrollment.  d) Presence of coexisting endocrine or immune system disorders.  e) Incomplete clinical documentation or diagnostic examinations. |
